# Induction of Antioxidant and Heat Shock Protein Responses During Torpor in the Gray Mouse Lemur, *Microcebus murinus*

**DOI:** 10.1016/j.gpb.2015.03.004

**Published:** 2015-06-17

**Authors:** Cheng-Wei Wu, Kyle K. Biggar, Jing Zhang, Shannon N. Tessier, Fabien Pifferi, Martine Perret, Kenneth B. Storey

**Affiliations:** 1 Institute of Biochemistry & Department of Biology, Carleton University, Ottawa, ON K1S 5B6, Canada; 2 UMR 7179 Centre National de la Recherche Scientifique, Muséum National d’Histoire Naturelle, Brunoy 91800, France; 3 Department of Biology, Genetics Institute, University of Florida, Gainesville, FL 32611, USA; 4 Biochemistry Department, Schulich School of Medicine and Dentistry, Western University, London, ON N6A 5C1, Canada; 5 Chemistry and Chemical Engineering Department, Royal Military College Of Canada, Kingston, ON K7K 7B4, Canada; 6 Department of Surgery & Center for Engineering in Medicine, Massachusetts General Hospital & Harvard Medical School, Charlestown, MA 02129, USA

**Keywords:** Heat shock proteins, Antioxidant capacity, Primate hypometabolism, Stress response

## Abstract

A natural tolerance of various environmental stresses is typically supported by various cytoprotective mechanisms that protect macromolecules and promote extended viability. Among these are antioxidant defenses that help to limit damage from reactive oxygen species and chaperones that help to minimize protein misfolding or unfolding under stress conditions. To understand the molecular mechanisms that act to protect cells during primate torpor, the present study characterizes antioxidant and heat shock protein (HSP) responses in various organs of control (aroused) and torpid gray mouse lemurs, *Microcebus murinus*. Protein expression of HSP70 and HSP90α was elevated to 1.26 and 1.49 fold, respectively, in brown adipose tissue during torpor as compared with control animals, whereas HSP60 in liver of torpid animals was 1.15 fold of that in control (*P* < 0.05). Among antioxidant enzymes, protein levels of thioredoxin 1 were elevated to 2.19 fold in white adipose tissue during torpor, whereas Cu–Zn superoxide dismutase 1 levels rose to 1.1 fold in skeletal muscle (*P* < 0.05). Additionally, total antioxidant capacity was increased to 1.6 fold in liver during torpor (*P* < 0.05), while remaining unchanged in the five other tissues. Overall, our data suggest that antioxidant and HSP responses are modified in a tissue-specific manner during daily torpor in gray mouse lemurs. Furthermore, our data also show that cytoprotective strategies employed during primate torpor are distinct from the strategies in rodent hibernation as reported in previous studies.

## Introduction

Survival in the face of unfavourable environmental conditions is a challenge for most animals. For instance, animal fitness is often limited by fluctuations in the availability of basic nutrients as well as by abiotic stresses (too hot, too cold, or too dry climate, low oxygen, *etc.*). When faced with environmental stresses, many animals exhibit adaptive responses that provide cytoprotection to combat potential damage to cells [1]. Changes in ambient temperature are among the most common stressors experienced by animals, which can often disrupt metabolic homeostasis. Such disruption can occur via a number of mechanisms including direct temperature effects on enzyme properties, protein conformation, and lipid fluidity, as well as secondary consequences such as changes in reactive oxygen species (ROS) generation. Many animals that must deal with extreme changes in temperature on a seasonal basis use strong metabolic rate depression to enter a torpid or dormant state when temperature is too cold (or too hot). They couple metabolic rate depression with enhanced cytoprotection, such as elevated levels of chaperones that help stabilize protein structure/function, as well as antioxidant defenses to deal with oxidative stress while in the hypometabolic state [2–4].

One of the hallmark responses to high temperature stress is the induction of heat shock proteins (HSPs), a group of chaperone proteins that function to aid proteome stability [5]. However, HSPs are now well known to be induced by many abiotic stresses that disrupt the cellular proteome, such as hypoxia, ischemia, oxidative stress, heavy metals, UV radiation, and low temperature [6,7]. HSP protein family members are named according to their molecular weight and the best known HSP proteins include HSP27, HSP40, HSP60, HSP70, HSP90α, and HSP110. Moreover, the family now includes many other chaperone proteins [8,9]. Although different HSPs respond to different cellular cues, their primary function is to maintain proteome stability, by guiding the folding of nascent proteins, re-folding misfolded proteins, preventing protein aggregation, and directing the degradation of unstable proteins [10,11]. HSP induction is a known component of metabolic rate depression in many systems, supporting long-term survival in hypometabolic states including dormancy, torpor, aestivation, and diapause. For example, the expression of HSP10, 60, 90, and 110 was all upregulated in the hepatopancreas following 14 days of estivation in snails (*Otala lactea*) [7], whereas expression of HSP70 and HSP27 (and its phosphorylated form) was upregulated in skeletal muscle of hibernating bats (*Myotis lucifugus*) [12,13].

Entry into hypometabolic states can also cause fluctuations to aerobic metabolism, leading to altered ROS production and potential oxidative damages [14]. This is particularly prominent in mammalian hibernation, since two factors come into play. First, intermittent arousals from torpor necessitate a huge increase in oxygen uptake and consumption (with a proportional increase in ROS generation) to power the thermogenesis required to rewarm the body to euthermia. Second, to maintain fluidity of lipid fuel depots at the low body temperature (Tb) during hibernation requires an increase in their polyunsaturated fatty acid (PUFA) content, which is highly susceptible to lipid peroxidation [15,16]. Hence, antioxidant defenses are necessary during the hibernation. These are provided by both low molecular weight metabolic antioxidants as well as antioxidant enzymes including superoxide dismutase (SOD), catalase, peroxiredoxin (PRX), thioredoxin (TRX), glutathione peroxidase, and other glutathione-linked enzymes [17,18].

Recent studies have presented the gray mouse lemur (*Microcebus murinus*) as a new model for the study of primate adaptation to environmental stress [19]. These small primates utilize daily or multi-daily torpor, reducing their metabolic rate (a maximum of ∼ 80% reduction compare to resting metabolic rate recorded) in order to cope with unfavourable conditions during the dry season in Madagascar, when food and water are limited and ambient temperatures are reduced [19]. Previous studies have shown that under short-day conditions combined with food restriction, gray mouse lemurs showed evidence of higher oxidative stress associated with increased torpor expression [20]. To date, little is known about the cytoprotective responses of lemurs during torpor. We hypothesized that during torpor, lemurs activate endogenous defense mechanisms to alleviate cellular stress, potentially using similar mechanisms as observed during torpor in well-studied mammalian hibernators (*e.g.*, bats and ground squirrels) [12,13,21–23]. To test this hypothesis, we examined the expression of proteins involved in the heat shock response and antioxidant defense in lemurs during daily torpor to identify potential molecular mechanisms of the stress response in primate torpor.

## Results

### Expression of HSPs during torpor

**Figure 1:**
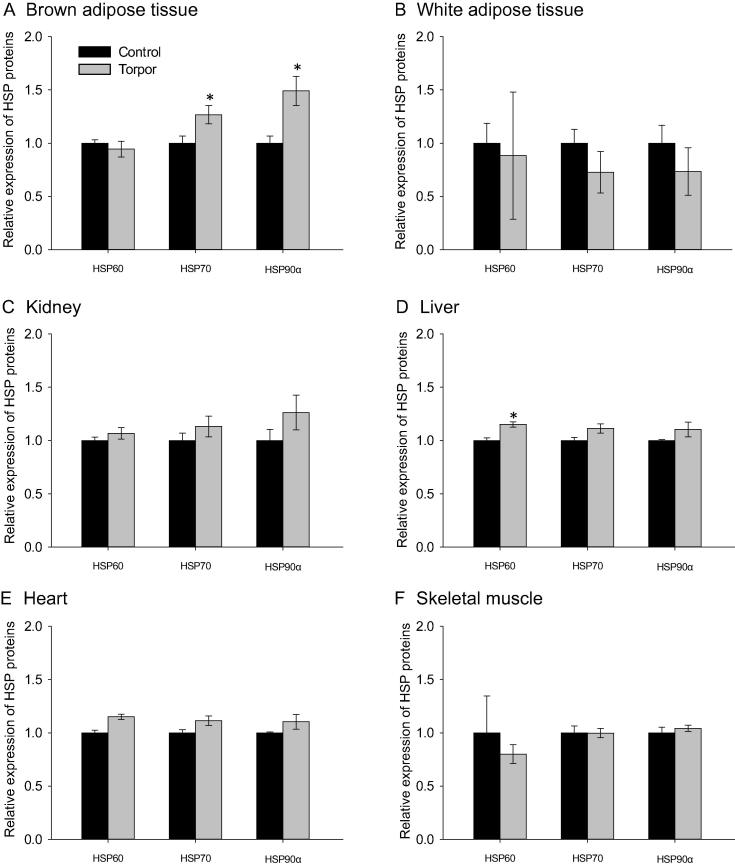
**HSP expression in gray mouse lemurs during daily torpor** Protein expression levels of HSP60, HSP70, and HSP90α were measured in different tissues, comparing control (aroused) and torpid lemurs. Studied tissues include brown adipose tissue (**A**), white adipose tissue (**B**), kidney (**C**), liver (**D**), heart (**E**), and skeletal muscle (**F**). All data were obtained by multiplex analysis using a Luminex 100 instrument and analyzed with Milliplex analyst software. Shown are histograms of median fluorescent intensity (MFI) of immune-reactive multiplex beads ± SEM (*n* = 4 independent trials for different animals). Data were analyzed using a two-tailed Student’s *t*-test; asterisk denotes significant difference from control (*P* < 0.05).

We first examined the expression of three major heat shock proteins (HSP60, HSP70, and HSP90α) in control (aroused) and torpid animals. Multiplex assay was employed to evaluate the protein expression in lemur tissues including the liver, muscle, heart, kidney, white adipose tissue (WAT), and brown adipose tissue (BAT). As shown in Figure 1, expression of HSP70 and HSP90α in BAT was significantly higher during torpor as compared to control animals; which was 1.27 ± 0.08 fold and 1.49 ± 0.14 fold, respectively (*P* < 0.05) (Figure 1A). Significantly higher amount of HSP60 was only observed in the liver during torpor (1.15 ± 0.02 fold, compared to control; *P* < 0.05) (Figure 1D). Otherwise, the expression of HSPs were comparable between control and torpor states in the WAT (Figure 1B), kidney (Figure 1C), heart (Figure 1E), and skeletal muscle (Figure 1F).

### Total antioxidant defense during torpor

**Figure 2:**
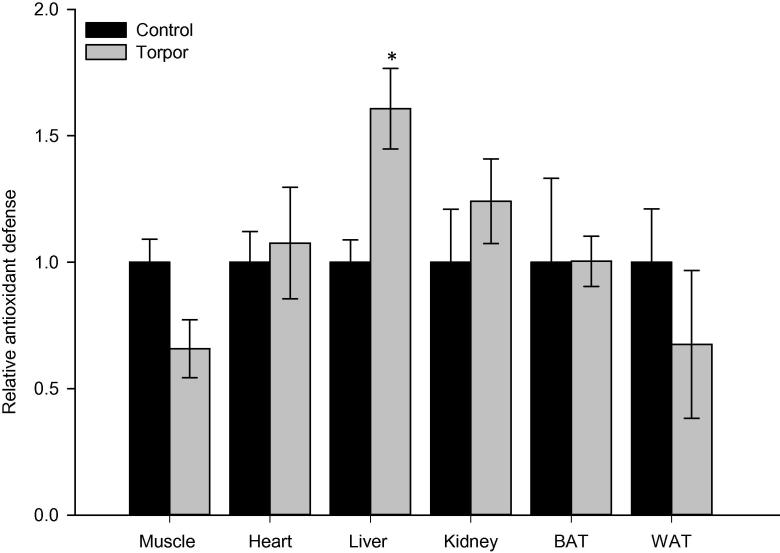
**Total antioxidant capacity in gray mouse lemurs during daily torpor** Antioxidant capacity measured was converted to Trolox equivalents against a Trolox standard curve in six tissues from control and torpid lemurs. Data are shown as means ± SEM (*n* = 4 independent trials from different animals). Data were analyzed using a two-tailed Student’s *t*-test; asterisk denotes significant difference from control (*P* < 0.05).

We then evaluated the total antioxidant capacity in six lemur tissues comparing control and torpor conditions (Figure 2). The antioxidant assay kit measures the cumulative antioxidant capacity supplied by a variety of cellular antioxidant molecules including vitamin C, vitamin E, glutathione, bilirubin, albumin, and uric acid. This is accomplished by measuring the rate at which these cellular antioxidant molecules inhibit the metmyoglobin-catalyzed oxidation of 2,2′-azino-bis (3-ethylbenzthiazoline-6-sulfonic acid (ABTS) to its radical cation form. A significant change in tissue antioxidant capacity was observed only in liver, which is 1.61 ± 0.16 fold in liver of torpid lemurs relative to control (aroused) animals (*P* < 0.05). Total antioxidant capacity did not change significantly between control and torpid lemurs in any of the other tissues, although antioxidant capacity in skeletal muscle tended to be lower during torpor.

### Expression of antioxidant enzymes during torpor

**Figure 3:**
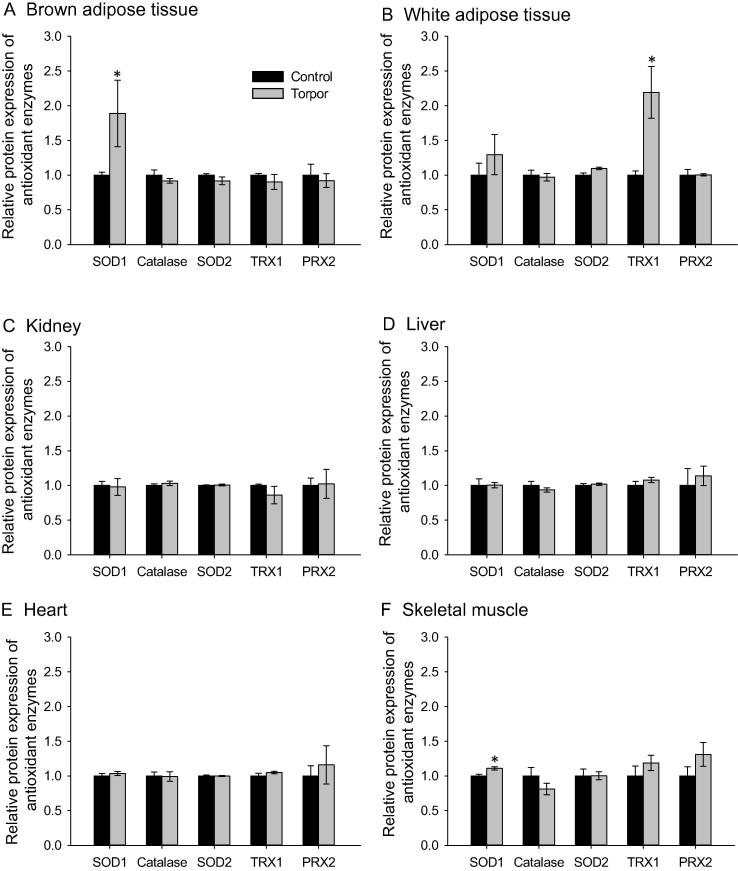
**Expression of antioxidant enzymes in gray mouse lemurs during daily torpor** Protein expression of Cu/Zn-SOD1 (SOD1), catalase, mitochondrial Mn-SOD (SOD2), thioredoxin 1 (TRX1), and peroxiredoxin 2 (PRX2) were measured in different tissues from control and torpid lemurs including brown adipose tissue (**A**), white adipose tissue (**B**), kidney (**C**), liver (**D**), heart (**E**), and skeletal muscle (**F**). All data were obtained by multiplex analysis using a Luminex 100 instrument and analyzed with Milliplex analyst software. Shown are histograms of median fluorescent intensity (MFI) of immune-reactive multiplex beads ± SEM (*n* = 4 independent trials from different animals). Data were analyzed using a two-tailed Student’s *t*-test; asterisk denotes significant difference from control (*P* < 0.05).

The protein expression levels of five antioxidant enzymes were measured using a Human Oxidative Stress Luminex panel in the six lemur tissues, comparing control (aroused) and torpor states. The five antioxidant enzymes measured in this study are involved in the detoxification of ROS molecules and are crucial to the oxidative stress response. These enzymes include Cu/Zn-SOD1 (the cytoplasmic form), Mn-SOD2 (the mitochondrial form), catalase, thioredoxin 1 (TRX1), and peroxiredoxin 2 (PRX2). Interestingly, expression of the majority of enzymes were comparable between torpor and arousal in most of the tissues examined (Figure 3). Among them, SOD1 the protein expression of cytoplasmic SOD1 was significantly higher in both BAT and skeletal muscle during torpor (Figure 3A and F), which was 1.9 ± 0.47 and 1.1 ± 0.02 fold as compared to control, respectively (*P* < 0.05). In addition, the expression of TRX1 was significantly higher during torpor (2.19 ± 0.37 fold as compared with control) in WAT (Figure 3B; *P* < 0.05).

## Discussion

To survive in challenging environments, animals often need to display considerable phenotypic plasticity at a metabolic level to adjust their energy demands to the realities of fuel/energy availability in the environment. To date, it has been well documented that coordinated reductions in energy expenditures on nonessential metabolic processes and a shift toward an altered metabolism that includes multiple cytoprotective mechanisms are hallmarks of stress-induced hypometabolism [1].

The present study focuses on these two classes of cytoprotective proteins to analyze their roles in lemur torpor. Interestingly, we found that expression of HSPs was mostly unchanged during torpor, with significant upregulation of selected HSPs observed only in BAT and liver (Figure 1). Elevated expression of HSP70 and HSP90α in BAT is particularly interesting, since this tissue produces heat through non-shivering thermogenesis (NST) to rewarm animals during arousal back to euthermia [24–27]. Previous studies have shown that gene and protein expression of HSP70 was upregulated in BAT of Sprague–Dawley rats during cold exposure, in parallel with the induction of uncoupling protein 1 (UCP1), suggesting a specific role for HSPs in this thermogenic organ [28]. Thermogenesis in BAT arises from uncoupling ATP synthesis from the electron transport chain in the mitochondria, which requires the expression of UCP1 [25–28]. In lemurs, expression of UCP1 is also upregulated in BAT to support NST during torpor and/or arousal [25]. HSP70, along with HSP90α, also functions as a molecular chaperone in the mitochondria to promote translocation and folding of mitochondrial proteins [29,30]. Upregulation of HSP70 and HSP90α could contribute to ensuring proper folding of UCP1 in the mitochondria, as an aid to NST during torpor in the lemur [28,29], and/or aid overall maintenance of the active conformations of proteins in the face of rapidly-rising temperatures in BAT during active NST.

Compared to the other tissues studied, expression of HSP60 was significantly upregulated only in the liver during torpor, albeit to a minor extent (Figure 1). HSP60 is a mitochondrial chaperone and plays a crucial role in regulating cell survival in response to increased levels of iron-dependent oxidative stress [31]. Previous studies showed that peroxide levels were elevated in HSP60-depleted cells, while elevated expression of HSP60 led to greater cellular resistance against H_2_O_2_ and superoxide anions [31]. Additionally, recent studies have also shown that upregulation of HSP60 expression is linked to chemically-induced ROS elevation in *Drosophila*, as well as type-2 diabetes associated oxidative stress in HeLa cells [32,33]. Therefore, the upregulation of HSP60 in lemur torpor could function similarly in regulating ROS resistance. The expression of HSP60 is also elevated during hibernation of ground squirrels, with previous microarray screening studies showing putative up-regulation of HSP40, HSP60, and HSP70 in liver during torpor [34]. Although the exact role of HSP60 in regulating oxidative stress is not fully understood, this link is not surprising due to the role of HSP60 in regulating mitochondrial protein import and folding [35].

To better understand the state of oxidative stress in lemur tissues during torpor, the total antioxidant capacity of six tissues was measured, along with expression levels of five antioxidant enzymes. An increase in total antioxidant capacity was observed in liver during torpor, but there were no significant changes in other tissues including BAT. The increase in liver antioxidant capacity may be indicative of a potential increase in oxidative stress during torpor. Interestingly, such increased antioxidant capacity was correlated with the elevated HSP60 expression, which was also seen in liver during torpor. Recent studies have also shown that the protein expression of SOD1 and catalase are elevated in some of tissues during hibernation of ground squirrels as compared to euthermic controls [21,36,37]. We observed limited changes in the protein levels of antioxidant enzymes across the six tissues during lemur torpor, with significant upregulation observed only for TRX1 in WAT and SOD1 in skeletal muscle and BAT. The general lack of change in the protein expression levels of antioxidant enzymes was intriguing; however, it should be noted that the antioxidant capacity of the tissue is the real measure of their functionality during torpor.

## Conclusion

The data presented in this study show that selected similarities in cytoprotective mechanisms occur between primate and rodent torpor, for example, activation of HSPs such as HSP60 in BAT and HSP70 in liver. However, in terms of antioxidant response, it appears that the transcriptional activation and increased synthesis of antioxidant enzymes are not the major responsive events in lemur torpor. This is in contrast to previous findings in ground squirrel torpor, with evidence of upregulation of PRX in the BAT and the heart during torpor, catalase in the skeletal muscle, and both SOD1 and SOD2 in BAT in response to torpor [21,38]. It is likely that the difference in duration and depth of torpor could differentially influence the transcriptional responses observed between torpid lemurs and hibernating ground squirrels. Ground squirrel torpor bouts can last for 3–25 days during the hibernation season, whereas lemur average daily torpor is just 8–15 h [19,39]. The shorter length of metabolic depression in lemur torpor could suggest that other more rapidly-activated mechanisms may be adapted in torpor. Such mechanisms of adaptation may include posttranslational modifications to proteins/enzymes, as is also known for reversible protein phosphorylation in rodent hibernation [40]. In conclusion, our study provides an initial insight into the molecular profiles of the stress response during primate torpor and provides a basis for the future exploration into the cellular mechanisms that are utilized primates to coordinate either daily torpor or seasonal hibernation.

## Materials and methods

### Animal treatments

A total of 8 female mouse lemurs (2–3 years of age) were used in the experiment. Animals were born in an authorized breeding colony at the National Museum of Natural History (Brunoy, France; European Institution Agreement No. D91-114-1). Protocols used for animal experiments were as described previously, and were carried out by Dr. Martine Perret and the Adaptive Mechanisms and Evolution Team [24,25]. Detailed animal protocols can be found in [41].

### Total protein lysate preparation

Sample lysates were prepared according to the manufacturer’s protocol for the assay panels used (Luminex, Toronto ON, Canada). Briefly, tissue samples of ∼50 mg were homogenized 1:2 (w/v) with a Dounce homogenizer using the supplied lysis buffer with the addition 1:100 (v/v) protease inhibitor cocktail (Catalog No. PIC003.1, Bioshop, Burlington ON, Canada). Supernatants containing soluble proteins were removed after centrifugation at 4500 × *g* for 15 min, and protein concentrations were determined by the Bradford assay. Lysates were normalized to the same concentration and diluted with manufacturer’s assay buffer to a final concentration of 0.6 μg/μl for the oxidative stress panel and to 4.5 ng/μl for the heat shock protein panel.

### Luminex multiplex assay

The multiplex immunoassays utilized for this study included the Human Oxidative Stress Magnetic Bead Panel (Catalog No. H0XSTMAG-18K, Millipore, Etobicoke ON, Canada) and the Heat Shock Protein Magnetic Bead 5-Plex Kit (Catalog No. 48-615MAG, Millipore). Luminex assays were performed as instructed by the manufacturer’s protocol, which were described in detail by Biggar et al. [41] in this special issue.

### Antioxidant capacity assay

Total antioxidant capacity was measured in control and torpid lemurs using an Antioxidant Assay kit (Catalog No. 709001, Cayman Chemicals, Ann Arbor, MI, USA). This assay determines total cellular antioxidant levels by measuring the rate at which antioxidants in each sample inhibit the metmyoglobin-catalyzed oxidation of 2,2′-azino-bis (3-ethylbenzthiazoline-6-sulfonic acid (ABTS) to its radical cation form. Frozen tissue samples were homogenized at 1:4 (w/v) in chilled antioxidant assay buffer with the addition of protease inhibitor as per manufacturer’s instructions. Samples were then centrifuged at 10,000 × *g* for 10 min at 4 °C. The resulting supernatants were collected and soluble protein was determined using the Bradford assay. All samples were standardized to the same protein concentration for the following assays. The antioxidant assays were initiated by addition of sample lysate along with metmyoglobin and chromogen as per the manufacturer’s protocol. Antioxidant capacity was then measured at 750 nm and converted to Trolox equivalents (mM/mg wet mass) using a Trolox antioxidant assay standard curve. Total Trolox equivalents were subsequently converted to relative antioxidant levels, by standardizing against the first control lemur sample.

### Statistical analysis

Data were presented as mean ± SEM (*n* = 4). All statistical and graphing analyses were performed using Sigmaplot 12.0 software. Statistical test was performed using the two-tailed Student’s *t*-test, with a significance level of *P* < 0.05.

## Authors’ contributions

All authors contributed to the conception and design of the project and to the editing of the manuscript. MP and FP carried out the animal experiments; CWW, KKB, SNT, and JZ conducted biochemical assays. Data analysis and assembly of the draft manuscript was carried out by KBS, CWW, and KKB. All authors read and approved the final manuscript.

## Competing interests

The authors have declared no competing interests.
